# Impact of target site mutations and plasmid associated resistance genes acquisition on resistance of *Acinetobacter baumannii* to fluoroquinolones

**DOI:** 10.1038/s41598-021-99230-y

**Published:** 2021-10-11

**Authors:** Mostafa Ahmed Mohammed, Mohammed T. A. Salim, Bahaa E. Anwer, Khaled M. Aboshanab, Mohammad M. Aboulwafa

**Affiliations:** 1 Department of Microbiology and Immunology, Faculty of Pharmacy, Al Azhar University, Assiut Branch, Assiut, 71526 Egypt; 2 Department of Microbiology and Immunology, Faculty of Pharmacy, Ain Shams University, Al Khalifa Al Ma’moun St., Abbassia, Cairo, Egypt; 3 Faculty of Pharmacy, King Salman International University, Ras Sedr, South Sinai, Egypt

**Keywords:** Microbiology, Pathogenesis

## Abstract

Among bacterial species implicated in hospital-acquired infections are the emerging Pan-Drug Resistant (PDR) and Extensively Drug-Resistant (XDR) *Acinetobacter* (*A*.) *baumannii* strains as they are difficult to eradicate. From 1600 clinical specimens, only 100 *A. baumannii* isolates could be recovered. A high prevalence of ≥ 78% resistant isolates was recorded for the recovered isolates against a total of 19 tested antimicrobial agents. These isolates could be divided into 12 profiles according to the number of antimicrobial agents to which they were resistant. The isolates were assorted as XDR (68; 68%), Multi-Drug Resistant (MDR: 30; 30%), and PDR (2; 2%). Genotypically, the isolates showed three major clusters with similarities ranging from 10.5 to 97.8% as revealed by ERIC-PCR technique. As a resistance mechanism to fluoroquinolones (FQs), target site mutation analyses in *gyrA *and *parC* genes amplified from twelve selected *A. baumannii* isolates and subjected to sequencing showed 12 profiles. The selected isolates included two CIP-susceptible ones, these showed the wild-type profile of being have no mutations. For the ten selected CIP-resistant isolates, 9 of them (9/10; 90%) had 1 *gyrA*/1 *parC* mutations (Ser 81 → Leu mutation for *gyrA *gene and Ser 84 → Leu mutation for *parC *gene). The remaining CIP-resistant isolate (1/10; 10%) had 0 *gyrA*/1 *parC* mutation (Ser 84 → Leu mutation for *parC* gene). Detection of plasmid-associated resistance genes revealed that the 86 ciprofloxacin-resistant isolates carry *qnrA* (66.27%; 57/86), *qnrS* (70.93%; 61/86), *aac (6')-Ib-cr* (52.32%; 45/86), *oqxA* (73.25%; 63/86) and *oqxB* (39.53%; 34/86), while *qepA* and *qnrB* were undetected in these isolates. Different isolates were selected from profiles 1, 2, and 3 and *qnrS, acc(6,)-ib-cr, oqxA,* and *oqxB* genes harbored by these isolates were amplified and sequenced. The BLAST results revealed that the *oqxA* and *oqxB* sequences were not identified previously in *A. baumannii* but they were identified in *Klebsiella aerogenes* strain NCTC9793 and *Klebsiella pneumoniae*, respectively. On the other hand, the sequence of *qnrS,* and *acc(6,)-ib-cr* showed homology to those of *A. baumannii*. MDR, XDR, and PDR *A. baumannii* isolates are becoming prevalent in certain hospitals. Chromosomal mutations in the sequences of GyrA and ParC encoding genes and acquisition of PAFQR encoding genes (up to five genes per isolate) are demonstrated to be resistance mechanisms exhibited by fluoroquinolones resistant *A. baumannii* isolates. It is advisable to monitor the antimicrobial resistance profiles of pathogens causing nosocomial infections and properly apply and update antibiotic stewardship in hospitals and outpatients to control infectious diseases and prevent development of the microbial resistance to antimicrobial agents.

## Introduction

*A. baumannii* is a strictly aerobic bacteria, non-lactose-fermenter, Gram-negative, and an opportunistic pathogen, which causes hospital-acquired infections^1^. The infection caused by this organism is difficult to treat^2^ due to its resistance to different classes of antimicrobial agents that is attributed to the intrinsic resistance and the organism ability to acquire resistance determinants as a result of the its genome plasticity. This endless capacity of genetic variability is the reason behind the global emerging problem caused by this organism^3^.

Different acquired resistance mechanisms as a result of overuse by physicians or misuse of antibiotics by patients have been reported for this pathogen and therefore, causing it to be able to express PDR or extensively drug-resistant (XDR) phenotypes particularly among critically ill patients^4^. Fluoroquinolones (FQs) in the last four decades had shown good activity against *A. baumannii* isolates. However, resistance to these drugs has rapidly emerged^4^. FQs are a widely prescribed medication in Egypt, and quinolone resistance has jumped up sharply^5^. *A baumannii* is now non-susceptible to the greatest repertoire of antimicrobial agents, involving FQs, and Pan-Drug resistant (PDR) is often responsible for the miscarriage of antibiotic treatment^6^. Resistance to FQs is primarily caused by the spontaneous mutations of genes in the area of the quinolone resistance-determining region (QRDR), which includes DNA gyrase, and topoisomerase IV. Changes that affect the drug to exert its goal due to alterations in the drug target genes, DNA gyrase (*gyrA*) or Topoisomerase IV C (*parC*) subunits, have been related to high levels of resistance to FQs^7^.

However, Martinez has detected the prevalence of plasmid-associated fluoroquinolones resistance (PAFQR) genes more than two decades ago^8^. Despite their insufficiency to confer FQ resistance, PAFQR executes an important role in the procuration of resistance to FQ by facilitating the selection of additional chromosomal resistance mechanisms, leading to a high level of FQ resistance and enabling bacteria to become fully resistant^9^. Most importantly, PAFQR can spread horizontally among *A. baumannii*^10^. Three kinds of PAFQR determinants have been described; *qnr* which protects fluoroquinolones targets from inhibition^8^. Inactivation of fluoroquinolones by acetylation with the common aminoglycoside acetyltransferase *aac (6)-Ib-cr*^11^, and efflux pumps QepAB and OqxAB^12^ are among the resistance mechanisms exhibited by this organism. Few studies were published about the prevalence of PAFQR determinants among *A. baumannii* isolates^13^. Touati, 2008 reported the detection of *qnrA* in *A. baumannii* in Algerian hospitals for the first time^14^. Jiang, 2014 reported the detection of two *qnrB*-positive isolates of PAFQR in clinical isolates of *A. baumannii* from Henan hospital, China^15^. A publication from China has also reported the prevalence of *qnrB6* and *qnrS2*^13^. Many studies have recently reported the emergence of resistance of *A. baumannii* in Egypt, limited studies are available on the mechanisms responsible for the resistance of *A. baumannii* in Upper Egypt to fluoroquinolones. Therefore, this study aimed to determine the antibiotic-resistant pattern of *A. baumannii* recovered from nosocomial infection cases and FQs-resistant mechanisms as an attempt to control this life-threatening pathogen.

## Materials and methods

### Specimens collection

A total of 1600 specimens were collected from blood, respiratory tract, urinary tract, catheters, wounds, and skin between January 2014 and March 2019 from two university hospitals; Al Azhar university hospital and Assuit university hospital, Assuit governorate, upper Egypt. Different clinical isolates were recovered. The study was approved by the ethics committee (Ethics Committee ENREC-ASU-63) of faculty of pharmacy-Ain Shams university. Both informed and written consents were obtained from the patients after explaining the study purpose. Also, all methods were performed following the relevant guidelines and regulations. The isolates were identified by standard microbiological methods^16^ and as described below.

### Phenotypic and genotypic identification of *A. baumannii* isolates

All specimens were cultured on nutrient agar (Oxoid Limited, England) as a general medium to recover all bacterial pathogens. The recovered bacterial isolates were characterized using two selective media, MacConky agar (Oxoid Limited, England) to identify lactose fermenting from non-lactose fermenting species and Herellea agar (Himedia, India) for selective scoring of *Acinetobacter* spp. Incubation was done at 37 °C for 24 h. All collected clinical isolates were propagated and maintained by standard microbiologic techniques^16^. For phenotypic identification, single separate colonies were handled for qualitative conventional diagnostic tests for *A. baumannii*; including Gram staining. The typical isolates showed Gram-negative reaction, catalase, and citrate utilization positive, while oxidase and indole tests were negative^17^. For genotypic identification, genomic DNA was extracted using the GeneJET Genomic DNA Purification Kit (Thermo, USA, catalog No. K0721) and used for amplification of the intrinsic *bla*_OXA-51_-like gene. This gene is unique for *A. baumannii* as previously described^18^. *A. baumannii* ATCC 19,606 was used as a positive control.

### Antimicrobial susceptibility testing

Disk diffusion was carried out on 19 antimicrobial agents using the Kirby-Bauer disk diffusion method as recommended by the Clinical and Laboratory Standards Institute (CLSI)^19^. The antibiotic discs used for susceptibility testing were imipenem (IMP 10 μg), meropenem (MEM 10 μg), piperacillin (PRL 100 μg), piperacillin/tazobactam (TZP 100/10 μg), ampicillin/sulbactam (SAM 10/10 μg), ceftazidime (CAZ 30 μg), cefotaxime (CTX 30 μg), ceftriaxone (CRO 30 μg), cefepime (FEP 30 μg), amikacin (AMK 10 μg), tobramycin (TOP 10 μg), gentamicin (CN 10 μg), ciprofloxacin (CIP 5 μg), levofloxacin (LEV 5 μg), gatifloxacin (GAT 5 μg), trimethoprim/sulfamethoxazole (SXT 12.5/23.75 μg), tigecycline (TGC15 μg), doxycycline (DO 30 mcg) and colistin (CT 10 units). All antimicrobial discs were purchased from Oxoid (UK) except gatifloxacin discs were purchased from Himedia (India). MDR, XDR, and PDR phenotypes were identified as previously determined^20^.

### Molecular typing of CIP-resistant *A. baumannii* isolates

Investigation of clonal relationship and diversity of the recovered *A. baumannii* isolates was determined by molecular typing of the CIP-resistant isolates using ERIC-PCR^21^. Genomic DNA was extracted using the Genomic DNA Purification Kit (Thermo Fisher Scientific) according to the manufacturer’s instructions. ERIC-PCR was carried out using the ERIC-1 (5’-ATGTAAGCTCCTGGGGATTCAC-3’) and ERIC-2 (5’-AAGTAAGTGACTGGGGTGAGCG-3’) primers as previously described^21^. The PCR products were analyzed using agarose gel electrophoresis at 1.5% (w/v) agarose containing 0.5 mg/ml ethidium bromide that was subsequently visualized by UV transilluminator. ERIC-PCR dendrogram was constructed by the use of UPGMA clustering method, Bionumeric program version 7.6 (Applied Maths). The percentage of similarity among the 86 CIP-resistant *A. baumannii* isolates was calculated by the use of Jaccard's Coefficient^22^.

### Molecular characterization of FQs resistance mechanisms in *A. baumannii* isolates

#### Detection of target-site mutation in genomic DNA

Genomic DNA was extracted using the GeneJET Genomic DNA Purification Kit (Thermo, USA, catalog No. K0721).

A. **PCR screening of FQ-resistance target site mutation**
  *PCR condition* denaturation at 95 °C for 2 min, then 35 cycles of amplification as follows: denaturation at 95 C for 30 s, annealing for 30 s at primer set-specific temperatures, and extension at 72 °C for 1 min), a final extension at 72 °C for 5 min. The oligonucleotides used to amplify and for sequencing analysis are shown in Table 1^23^.
B. **Purification of PCR products**
  Purification of PCR products before sequencing was performed using the PCR purification kit (Thermo, USA, Catalog number: K0701). Briefly, 1 volume of DNA binding buffer was mixed with each volume of PCR product. The sample mixture was then transferred into thermo-spin and centrifuged. The column was washed with a DNA wash buffer. Finally, the column was eluted with 50 µl DNA elution buffer. The purified DNA was stored at -20^0^C for safe storage of amplicons till sequencing using Sanger sequencing technique (Applied Biosystems genetic analyzers (ThermoFisher, Uk)).
C. **Target site sequence analysis**
  A 613 bp and A 919 bp fragments of the corresponding *gyrA* and *parC* genes were amplified from 12 selected isolates (one isolate from one out of 12 profiles, isolates of the profiles 1 to 12 showed resistance to 19, 18, 17, 16, 15, 14, 13, 12, 11, 9, 8, 6 out of 19 tested antimicrobial agents, respectively. The chosen 12 profiles included PDR XDR and MDR isolates). Nucleotide sequences were visualized by SnapeGene Viewer (version 5.1.4.1 software 2020). ORFs were identified using the ORF finder tool. The sequences of *gyrA* and *parC* genes from the selected 12 isolates were compared to their corresponding ones of the *gyrA* and *parC* genes of *A. baumannii* ATCC 19,606 as previously described^24^. Pairwise codon-based nucleotide alignments (CDS-alignments) of the *gyrA* and *parC* genes from the selected isolates against their corresponding sequences of the standard strain were carried out.

**Table 1:** Oligonucleotides used to amplify and for sequencing analysis of fluoroquinolone-resistance determining regions.

Target gene	Primer	Oligonucleotide sequence (5` to 3`)	Expected amplicon size (bp)	Annealing temperature
*gyrA*	gyrA-F	TGCATTGCCGGATGTGAGA	613	57
	gyrA-R	ACCGGTACGGTAGGCATCAA		
*parC*	parC-F	CAGAAAACCGCTCTGTAGCC	919	
	parC-R	ACTGCTTCCGCATCAATAC		

#### Plasmid-associated fluoroquinolone resistance (PAFQR) genes

A. **Plasmid DNA extraction**
  Plasmid DNA was extracted and purified using the GeneJET Plasmid Miniprep Kit (Thermo, USA, catalog No. K0502). Briefly, a single colony from each test isolates was picked up from a freshly streaked selective plate to inoculate 1–5 mL of Mueller Hinton broth mixed with ciprofloxacin (0.125 mg/ml) in a test tube. The tubes were incubated at 37 °C for 12–16 h under shaking at 200–250 rpm in a shaker incubator. The volume of the container either a test tube or a flask was at least 4 times the culture volume. Cell pellets of the bacterial culture were collected by centrifugation at 6800 × g (8000 rpm) for 2 min using a micro-centrifuge. The supernatant was decanted and bacterial pellets were resuspended and subjected to alkaline lysis to liberate the plasmid DNA. The resulting lysate was neutralized to create appropriate conditions for the binding plasmid DNA on the silica membrane in the spin column. By centrifugation, cell debris was pelleted, and the supernatant containing the plasmid DNA was loaded onto the spin column membrane. The adsorbed DNA was washed to remove contaminants and then eluted with 50 µL of the elution buffer (10 mM Tris–HCl, pH 8.5). The extracted plasmid DNA was stored at − 20 °C for subsequent use**.**
B. **PCR screening of PAFQR determinant genes**
  Plasmid extracts of all isolates were screened for the following genes: *qnrA, qnrB, qnrS, aac(6′)-Ib-cr, qepA, oqxA ,* and *oqxB* using gene-specific primers listed in Table 2. PCR conditions and primer sequences were as previously described^25^.
C. **Sequencing of resulting amplicons of target genes**
  PCR amplicons of target genes: *qnrA, qnrB, qnrS, aac(6′)-Ib-cr, qepA, oqxA ,* and *oqxB* resulting from plasmid extracts of test isolates from selected profiles numbers 1, 2, and 3 only were sequenced using Sanger sequencing technique (Applied Biosystems genetic analyzers (ThermoFisher, UK)). Such kind of isolates might be more life-threatening ones.

**Table 2:** Oligonucleotide primers, their sequences, and annealing temperatures used for detection and sequencing of PAFQR genes, and the expected sizes of resulting amplicons.

Target genes	Primer	Oligonucleotide sequence (5` to 3`)	Expected amplicon size (bp)	Annealing temperature (°C)
*qnrA*	F	GCCCGCTTCTACAATCAAGT	347	60
	R	GGCAGCACTATTACTCCCAAG		
*qnrB*	F	TATGGCTCTGGCACTCGTT	193	
	R	GCATCTTTCAGCATCGCAC		
*qnrS*	F	TCGGCACCACAACTTTTCAC	255	
	R	TCACACGCACGGAACTCTAT		
*acc (6') Ib-cr*	F	CTTGCGATGCTCTATGAGTGG	480	
	R	GAATGCCTGGCGTGTTTGAA		
*qepA*	F	TCTACGGGCTCAAGCAGTTG	312	55
	R	ACAGCGAACCGATGACGAAG		
*oqxA*	F	CTCTCCTTTCTGCTCGTCGG	489	67
	R	AATAGGGGCGGTCACTTTGG		
*oqxB*	F	TAGTGCTGGTGGTGCTGGTA	480	68
	R	GGGTAGGGAGGTCTTTCTTCG		

### Statistical analysis

All data were analyzed using the GraphPad PRISM (Version.8.4.0.671). Descriptive statistics were used. The *p-value* < 0.05 was considered a statistically significant used Chi-Square test.

### Ethical approval

The study was approved by the Ethics Committee (Ethics committee ENREC-ASU-63) at Faculty of Pharmacy-Ain Shams University where both informed and written consent were obtained from the patient after explaining the study purpose.

## Results

### Phenotypic and genotypic identification of *A. baumannii* isolates

The isolates were identified according to standard microbiological methods^16^. The isolates showing characters suspected to be *Acinetobacter* species were subjected to some biochemical and growth conditions tests. The suspected isolates of *Acinetobacter* spp gave the following test results: negative reaction with oxidase, and indole, positive reaction with catalase test, growth at 44 °C, and positive for citrate utilization test. Examination of cell morphology of Gram-stained films revealed that: all *Acinetobacter* isolates were short, Gram-negative diplo-coccobacillus (sometimes de-staining of primary stain was difficult due to tendency of some isolates to retain crystal violet) with pairing or clustering arrangements. From a total of 623 non-lactose fermenters isolates, only 151 had these mentioned characters.

All *Acinetobacter* species candidates (151 isolates) were further subjected to conventional PCR to confirm their identity. The characteristic band at 353 bp for the *bla*_OXA-51_-like gene of *A. baumannii* was used for this identification and confirmation. From the total 151 isolates subjected to PCR, only 100 isolates were positive for *bla*_OXA-51_ and confirmed to be *A. baumannii* as shown in Supplementary Fig. S1.

The specimens that showed the highest percentage 61% (61/100) of *A. baumannii* contamination were obtained from the respiratory tract (ETT 29%, nasal 17%, sputum 13%, throat 2%), followed by urinary tract infection 17% (urine 9%; urinary tract catheter 8%) and blood 12%, while the lowest (2%; 2/100) was from skin and CVC.

### Antimicrobial susceptibility testing

As shown in Table 3 all *A. baumannii* isolates exhibited high resistance to most of the tested antimicrobial agents. However, the higher resistance was recorded to piperacillin (99%) and cephalosporins (98%). On the other hand, the most effective antimicrobial agents were recorded to be colistin (only 5% of isolates showed resistance) followed by doxycycline (only 57% of isolates showed resistance).

**Table 3:** Antibiogram analysis of the 100 recovered *A. baumannii* isolates.

Tested antibiotic	Number of isolates		*P* Value
	Sensitive	Resistant	
IMP	24	76	< 0.0001
MEM	18	82	
PRL	1	99	
TZP	6	94	
SAM	11	89	
CAZ	2	98	
CTX	2	98	
CRO	2	98	
FEP	2	98	
AK	23	77	
CN	21	79	
TOP	7	93	
CIP	14	86	
GAT	20	80	
LEV	17	83	
SXT	13	87	
CT	95	5	
TGC	2	98	
DO	43	57	

IMP: Imipenem, MEM: Meropenem, PRL: Piperacillin, TZP: Tazopactam/Piperacillin, SAM: Sulbactam /Ampicillin, CAZ: Ceftazidime, CTX; Cefotaxime, CRO: Ceftriaxone, FEP: Cefepime, AK: Amikacin, CN: Gentamicin, TOB: Tobramycin, CIP: Ciprofloxacin, GAT: Gatifloxacin, LEV: Levofloxacin, SXT: Sulfamethoxazole/Trimethoprim, CT: Colistin, TGC: Tigecycline, DO: Doxycycline. The *p*-value for the chi-square statistic for each tested antibiotic is < 0.0001, which is smaller than the alpha level of 0.05.

Resistance profile analysis of *A. baumannii* susceptibility results (100 isolates) revealed that two isolates (2%) were detected as pan drug-resistant (PDR), 68% isolates were extensive drug-resistant (XDR), and 30% isolates were multidrug-resistant (MDR).

**Figure 1:**
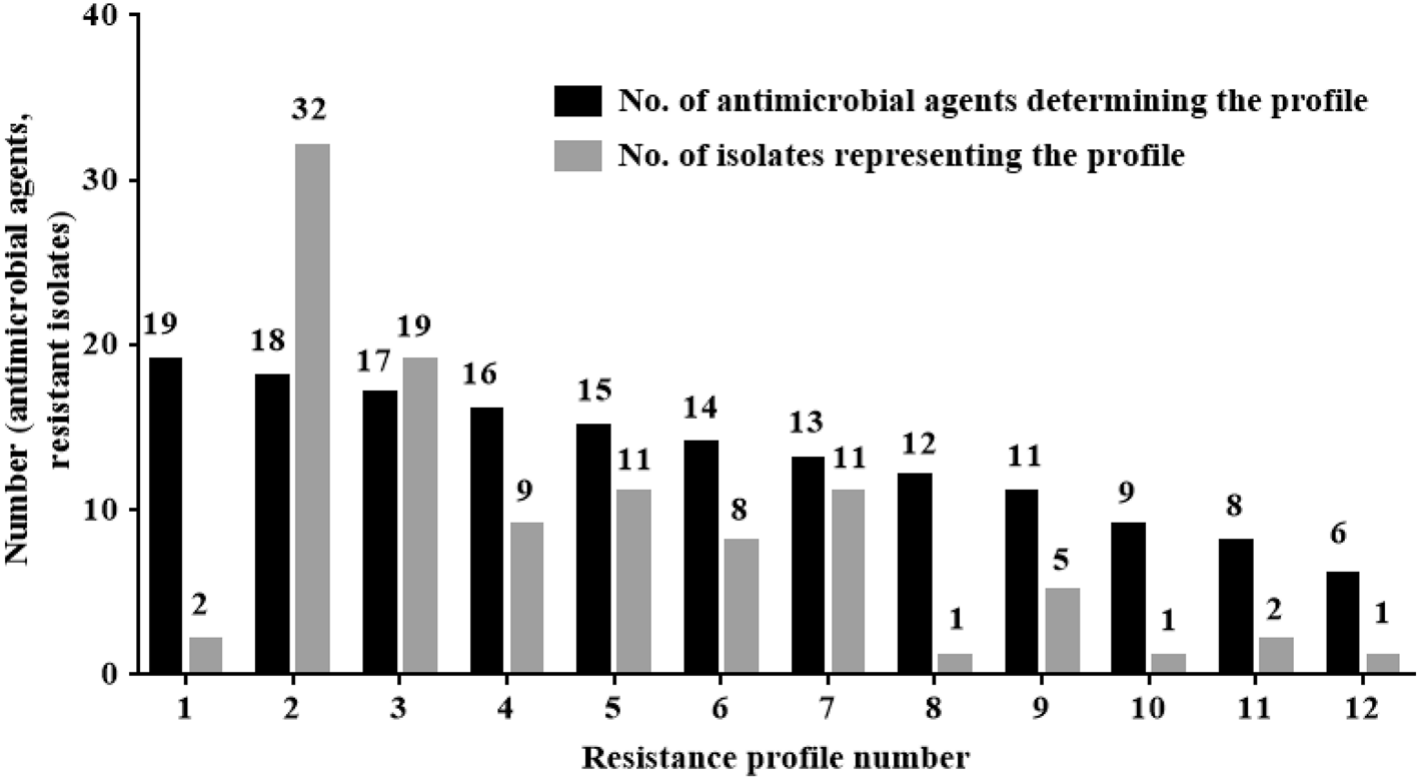
Resistance profiles of *A. baumannii* isolates against 19 tested antimicrobial agents and the number of isolates representing each profile.

Analyses of the resulting *A. baumannii* susceptibility to the 19 tested antimicrobial agents showed the diversity of isolates' resistance to the tested agents. They were divided into 12 profiles according to the number of antimicrobial agents to which they were resistant. The resistance scope ranged from 6 to 19 antimicrobial agents. The first profile is PDR (two isolates), which represents isolates resistant to 19 tested antimicrobial agents. The second profile is XDR (32 isolates), which represents isolates resistant to all tested antimicrobial agents except colistin, and the third profile (19 isolates) represents isolates resistant to 17 antimicrobial agents. On the other hand, profiles numbers 4 to 12 represent *A. baumannii* isolates susceptible to 3 to 13 of the 19 tested antimicrobial agents (Fig. 1).

### Molecular typing of CIP-resistant *A. baumannii* isolates

**Figure 2:**
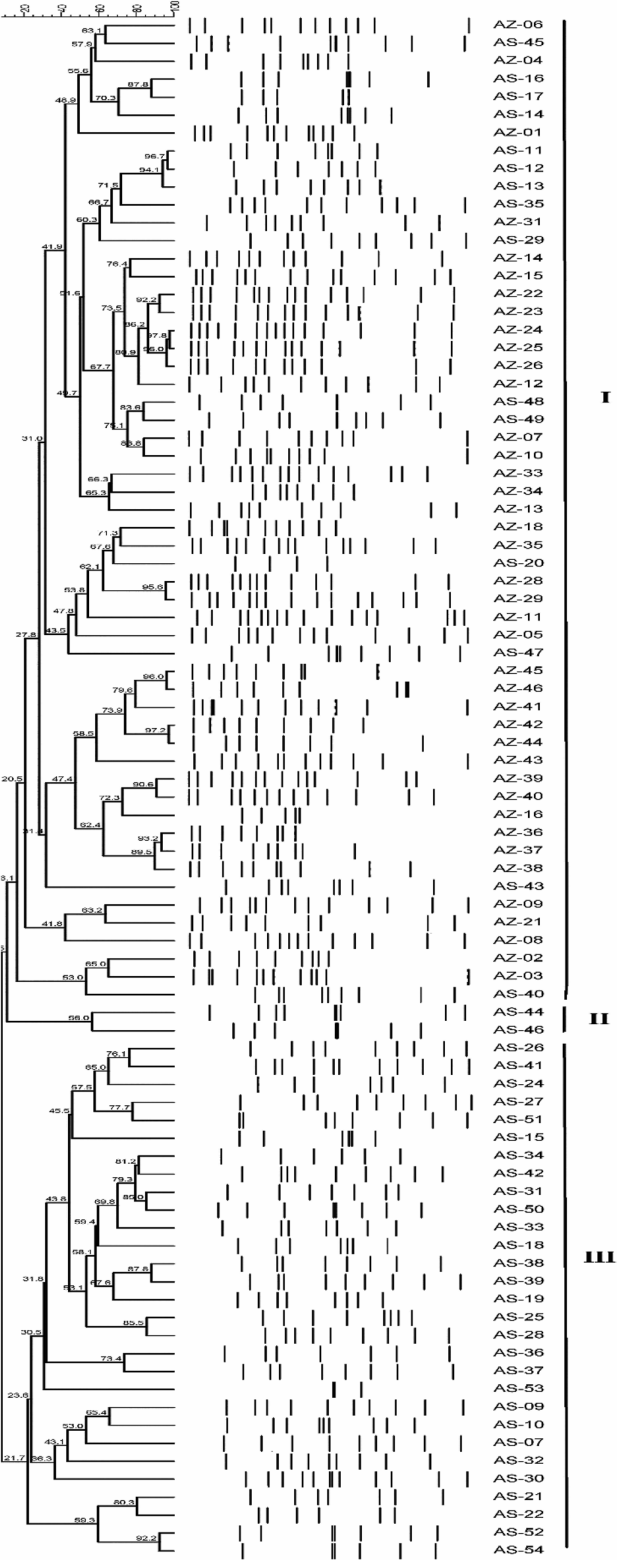
Dendogram clustering for 86 *A. baumannii* isolates resistant to CIP as determined by ERIC-PCR using UPGMA clustering method.

Determination of clonal relationship and diversity of hospital-acquired infection caused by *A. baumannii* isolates were carried out by the genotyping method using ERIC-PCR technique. This was conducted for all *A. baumannii* isolates resistant to CIP (n = 86), and the results were subjected to phylogenetic analysis (Fig. 2). As a result, the isolates could be divided into three major clusters (I, II, and III) with similarities ranging from 10.5 to 97.8%. The high diversity indicates multiple contamination sources with *A. baumannii.* Cluster I represents 63.95% (55/86). Cluster II represents 2.32%, and included only two isolates namely; AS-44 and AS-46, while cluster III constitutes 37% (29/86) of the total isolated *A. baumannii* (Fig. 2). All *A. baumannii* in clusters II and III were recovered from Assiut University while most isolates included in cluster I was recovered from Al-Azhar University.

### Molecular characterization of *A. baumannii* resistance mechanisms to FQs

#### Detection of target site mutation

**Figure 3:**
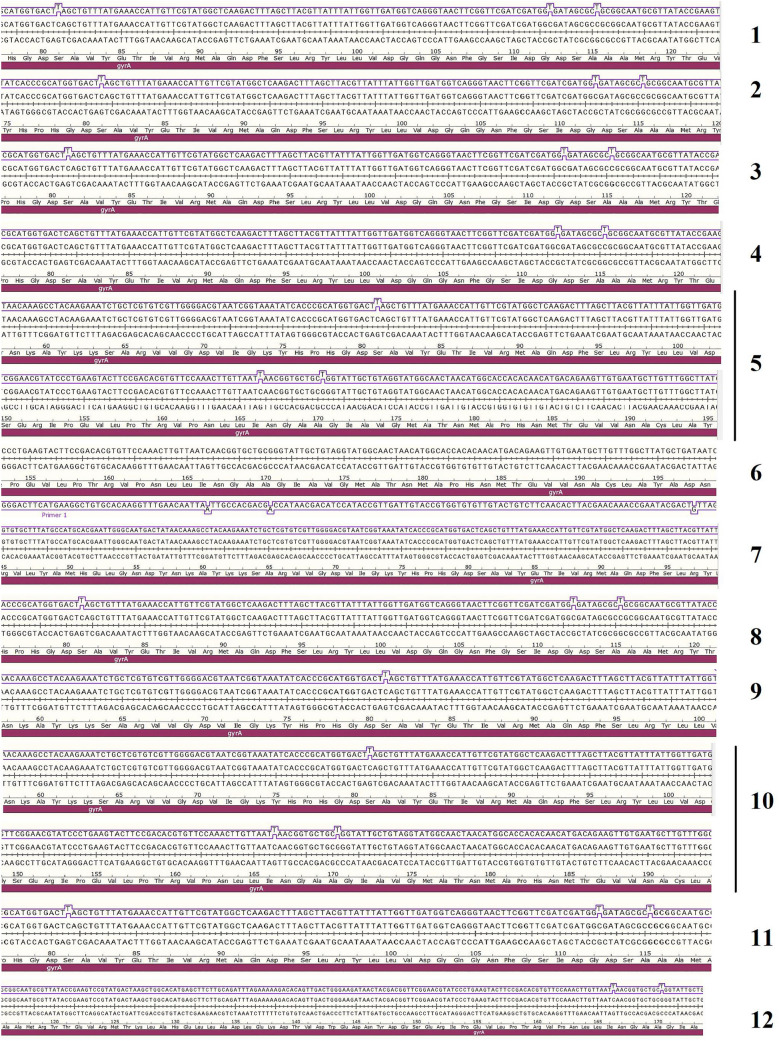
Pairwise alignment of nucleotide sequences (CDS region) of *gyrA* genes of 12 tested *A.baumannii* isolates versus the wild type gene of *A.baumannii* ATCC 19,606 retrieved from the GeneBank database using SnapGene Viewer software.

**Figure 4:**
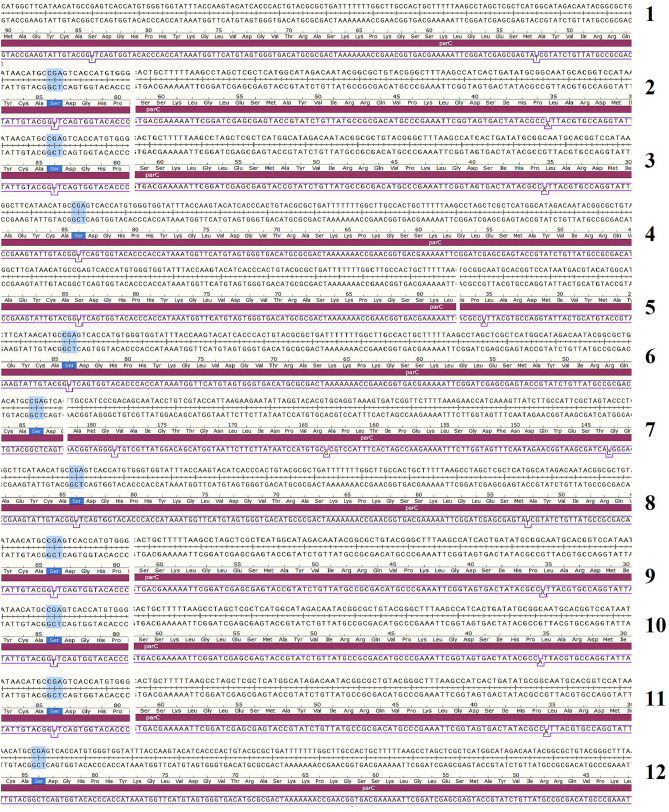
Pairwise alignment of nucleotide sequences (CDS region) of *ParC* genes of 12 tested *A.baumannii* isolates versus the wild type gene of *A.baumannii* ATCC 19,606 retrieved from the GeneBank database using SnapGene Viewer software.

Out of 12 phenotypic profiles (Fig. 1), one isolate from each profile was selected, and its purified genome was subjected to PCR amplification of *gyrA* and *parC* genes followed by sequencing of the resulting amplicons. Out of the 12 isolates, 10 isolates were fluoroquinolone-resistant, and two isolates were fluoroquinolone susceptible (AS-01 and AS-05). *gyrA* and *parC* gene sequences of the selected *A. baumannii* isolates were analyzed and they showed similarities ranging from 95 to 100% to their corresponding sequences deposited in GenBank nucleotide database under accession numbers shown in Supplementary Table 1. Target site mutation analyses in *gyrA* and *parC* gene sequences of the selected twelve *A. baumannii* isolates represent 12 profiles. Two isolates (susceptible ones to CIP) had a wild-type profile. For the ten isolates (CIP-resistant ones), 9 of them (9/10; 90%) had 1 *gyrA* and 1 *parC* mutations Ser 81 → Leu mutation for *gyrA* gene and Ser 84 → Leu mutation for *parC* gene. The remaining CIP-resistant isolate (1/10; 10%) had (0 *gyrA* /1 *parC*) mutation (Ser 84 → Leu mutation) for *parC* gene. All tested isolates had a silent mutation in one or more positions of either *gyrA* or *parC* or both *gyrA* and *parC* (Supplementary Table 2, and Figs. 3 and 4).

#### Plasmid-associated fluoroquinolone resistance (PAFQR) genes

**Figure 5:**
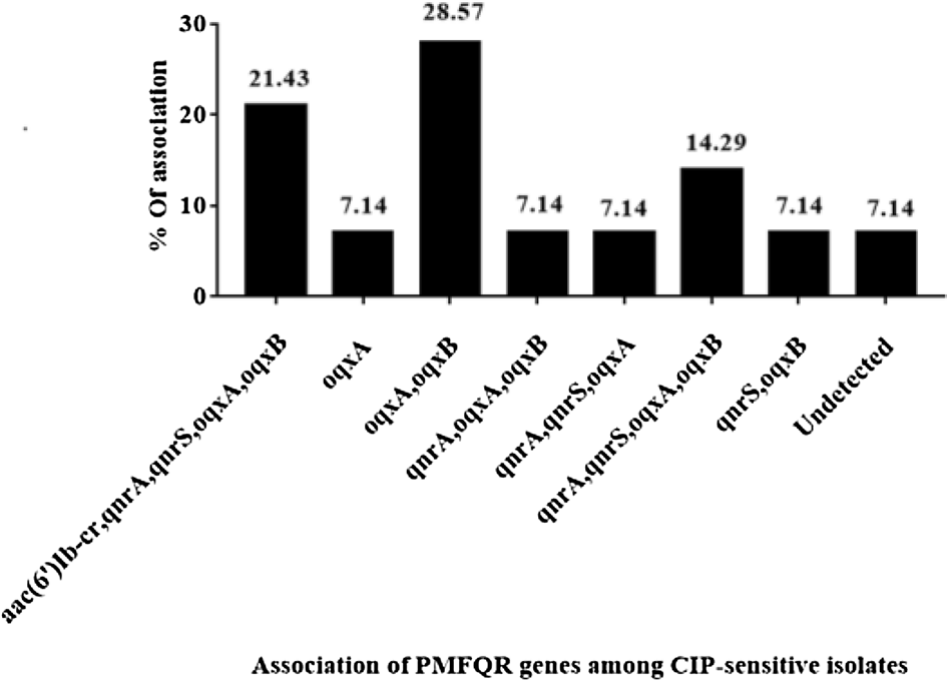
Distribution of PAFQR genes among CIP-sensitive *A. baumannii* isolates.

A. **DNA plasmid extraction**
  The variable number of bands per isolate may be plasmids with different molecular weights that were detected (no endonuclease digestion was used) in 99% of *A. baumannii*. Besides, no plasmid could be detected in 1% of the isolates.
B. **PCR screening *A. baumannii* isolates for PAFQR genes**
  All isolates were screened for PAFQR genes (*qnrA, qnrB, qnrS, acc(6)-ib, qepA, oqxA,* and *oqxB*) using conventional PCR. The expected sizes of PCR products mentioned in Table 2 were obtained**.** Ciprofloxacin-resistant isolates (86%; 86/100) carried *qnrA* (66.27%; 57/86), *qnrS* (70.93%; 61/86), *aac (6')-Ib-cr* (52.32%; 45/86), *oqxA* (73.25%; 63/86) and *oqxB* (39.53%; 34/86) resistance genes, while the resistance genes *qepA* and *qnrB* were undetected in these isolates. Although 14 isolates were susceptible to ciprofloxacin, some resistant genes were detected in these isolates, these included *qnrA* (7/14), *qnrS* (7/14), *aac (6')-Ib*-*cr* (3/14), *oqxA* (12/14), and *oqxB* (11/14)**.**
C. ***Distribution of PAFQR genes among A. baumannii isolates***
(i) **Among CIP-resistant isolates**
  Analysis of the 86 CIP-resistant isolates for their acquisition of PAFQR genes gave 26 profiles (Table 4). The prevalence of PAFQR genes was highly observed among 84 isolates. The plasmid extracts of isolates might contain up to five genes per isolate. On the other hand, the plasmid extract of one isolate (AS-29) did not show any resistance gene and another isolate (AZ-18) which did not carry any plasmid. The latter isolate was recovered from the sputum of an admitted patient who stayed for only three days in Al-Azhar university hospital.
(ii) **Among CIP-sensitive isolates (n = 14)**
  For the 14 CIP-sensitive isolates, analyses of occurrence and distribution of PAFQR genes in their corresponding plasmid extracts revealed that these plasmids harbor up to five genes, which gave 8 profiles of genes association (Fig. 5). On the other hand, one isolate did not harbor any PAFQR resistance gene.
(iii) ***PAFQR gene sequences analyses***
  PCR products of different genes from an isolate representing each profile 1, 2, and 3 were selected for sequencing (contain more life-threatening isolates), these included *qnrA, qnrS, acc(6,)-ib-cr, oqxA,* and *oqxB*. The amplicon sizes were 347, 255, 480, 489, and 480 bp, respectively. The BLAST of the NCBI (www.ncbi.nlm.nhi.gov) was used to search databases for detecting the similarity in nucleotides and amino acid sequences of these studied genes to those deposited in the databases. The BLAST results revealed that the *oqxA* and *oqxB* sequences were not identified previously in *A. baumannii* but they were identified in *Klebsiella aerogenes* strain NCTC9793 and *Klebsiella pneumoniae*, with an identity of 99.78% and 99.77%, respectively. On the other hand, the sequences of *qnrA, qnrS, acc(6,)-ib-cr,* and *oqxA,* showed homology to those of *A. baumannii* deposited in GeneBank database with identity ranged from 97.98 to 98.28% (Table 5).

**Table 4:** Distribution of PAFQR genes among CIP-resistant *A. baumannii* isolates.

Profile number	Detected PAFQR gene(s)	Association of PAFQR genes	Incidence	%*
1	0	Undetected	2	2.33
2	1	*oqxA*	1	1.16
3	1	*qnrA*	1	1.16
4	1	*qnrS*	2	2.33
5	2	*aac(6')Ib-cr, oqxA*	4	4.65
6	2	*aac(6')Ib-cr, oqxB*	1	1.16
7	2	*aac(6')Ib-cr, qnrA*	1	1.16
8	2	*oqxA, oqxB*	3	3.49
9	2	*qnrA, oqxA*	2	2.33
10	2	*qnrA, qnrS*	11	12.79
11	2	*qnrS, oqxA*	1	1.16
12	2	*qnrS, oqxB*	1	1.16
13	3	*aac(6')Ib-cr, oqxA, oqxB*	2	2.33
14	3	*aac(6')Ib-cr, qnrA, oqxA*	4	4.65
15	3	*aac(6')Ib-cr, qnrS, oqxA*	1	1.16
16	3	*aac(6')Ib-cr, qnrS, oqxB*	2	2.33
17	3	*qnrA, oqxA, oqxB*	1	1.16
18	3	*qnrA, qnrS, oqxA*	11	12.79
19	3	*qnrA, qnrS, oqxB*	1	1.16
20	3	*qnrS, oqxA, oqxB*	2	2.33
21	4	*aac(6')Ib-cr, qnrA, oqxA, oqxB*	3	3.49
22	4	*aac(6')Ib-cr, qnrA ,qnrS, oqxA*	12	13.95
23	4	*aac(6')Ib-cr, qnrA, qnrS, oqxB*	1	1.16
24	4	*aac(6')Ib-cr, qnrS, oqxA, oqxB*	7	8.14
25	4	*qnrA, qnrS, oqxA, oqxB*	2	2.33
26	5	*aac(6')Ib-cr, qnrA, qnrS, oqxA, oqxB*	7	8.14

*% was calculated relative to total CIP-resistant *A. baumannii* isolates (n = 86).

**Table 5:** The PAFQR gene amplicons sequences of some selected *A. baumannii* isolates and their identity to sequences deposited in GeneBank database.

Gene^(a)^	GeneBank accession no. for the deposited studied sequence	Strain showing sequence homology to the studied sequence	% of the identity of the studied sequence to its homology in database^(b)^	Profile
*qnrS1*	KY640596.1	*Acinetobacter baumannii* strain E162ABMO	98.28	1, 2, 3
*acc(6)-ic-cr*	CP040425.1	*Acinetobacter baumannii* strain PB364	97.98	2, 3^(c)^
*oqxA*	LR134280.1	*Klebsiella aerogenes* strain NCTC9793	99.78	1, 2, 3
*oqxB*	CP023134.2	*Klebsiella pneumoniae*	99.77	2, 3^(c)^

The level of significance for the % of identity of the studied sequence to its homology in database was mentioned.

^(a)^no successful sequence of *qnrA* could be obtained although its amplicon was sent twice to different laboratories.

^(b)^at *p*-value ≤ 0.05 level of significance.

^(c)^these genes gave negative results with profile number 1 isolate.

## Discussion

*A. baumannii* is a Gram-negative bacterium, can withstand a wide range of environmental conditions and also can survive on surfaces. These characters enable it to be implicated in many nosocomial infections and outbreaks. It is a strict aerobic organism^2^. The infection caused by *A. baumannii* is difficult to treat^2^. It has been recognized by the Infectious Disease Society of America as one of the six highly drug-resistant hospital pathogens^26^.

Antimicrobial agents use strategies are known to significantly reduce the frequency of bacterial infections in patients^27^. Among which fluoroquinolones (FQs) have the widest use and are currently recommended by different physicians as they have multiple applications and different advanced generations^4^. Nevertheless the emergence and spread of bacterial resistance to FQs among Gram-negative bacteria generally and *A. baumannii* specifically is becoming increasingly serious with their extensive use. FQs in the last forty years had shown good activity against *A. baumannii* isolates, However, resistance to these drugs has rapidly emerged^15^. FQs are widely prescribed medication in Egypt, and resistance to FQs has skipped pointedly^5^. The developing resistance of *A. baumannii* to antimicrobial agents has been described and this was attributed to the abundance of these antibiotics in multiple pharmaceutical markets^28^, besides their misuse^29^. *A. baumannii* infection is difficult to remedy, as of its everlasting fullness to acquire antimicrobial resistance due to the suppleness of its genome^30^. Many acquired resistance mechanisms have been reported for this pathogen and therefore, render it able to express MDR, XDR, or PDR phenotypes that were associated with significant morbidities and mortalities^4^. Therefore, this study aimed to determine the mechanisms behind *A. baumannii* resistance to FQs. For achieving this aim, the following objectives were studied: (i) the antibiotic-resistant phenotypes of *A. baumannii* recovered from nosocomial infection cases in Al-Azhar university hospital and Assiut university hospitals, both in Assiut governorate Upper Egypt; (ii) studying the molecular mechanisms responsible for the resistance of *A. baumannii* isolates to FQs which included target site mutation and ESBLs as well as PAFQR genes acquisition.

In this study, a total of 1600 specimens were collected from different clinical hospitalized patients of two major university hospitals in Upper Egypt during the period between January 2014 and March 2019. Out of 151 *Acinetobacter* candidate isolates subjected to PCR, only 100 isolates were positive for *bla*_OXA-51_ and confirmed to be *A. baumannii.* The identification of *A. baumannii* phenotypically is difficult due to significant phenotypic overlapping with other species, which are genotypically closely related to each other^31^**.**

The study results revealed that the highest number of *A. baumannii* isolates was recovered from respiratory and urinary tracts as well as blood specimens indicating the involvement of this pathogen in infection of these sites. The 100 recovered *A. baumannii* isolates comprised 61 isolates from respiratory tract infection {Enotreacheal tubes (29), nasal (17), sputum (13), and throat (2)}, blood infection (12), and urinary tract infections (9). It has been reported^32^ that the respiratory tract, blood, and urinary tract constitute the most predominant sources of *A. baumannii* pathogen. *A. baumannii* was also recovered from wounds or soft-tissue infections (6), skin (2), catheter-associated infections {central venous catheter (2), and urinary tract catheter (8)} in agreement with the results reported by^33^. Worldwide, it was reported that *A. baumannii* infection differs according to both the anatomical site and the clinical conditions of the patients^34^.

FQs in the last four decades had shown good activity against *A. baumannii* isolates, however, resistance to these drugs has quickly been rised^15^. FQs are a widely prescribed medication in Egypt, and FQs resistance has jumped up sharply^4, 5, 35^. Our findings emphasize that most *A. baumannii* isolates (71%) were recovered from patients treated for long period with FQs, while the rest (29%) of *A. baumannii* isolates were recovered from patients treated with other antimicrobial agents such as cephalosporins, imipenem, and the penicillin derivative (amoxicillin) or combination (amoxicillin-clavulanic acid). Un-rational use, low dose, and misuse of antimicrobial agents (empirical antibiotcs use by non hospitalized patients) result in the development of microbial resistance and so increase the risk of nosocomial *Acinetobacter* infections in hospitals^4^.

The increase in *A. baumannii* resistance constitutes a global issue^36^. Every year, the life of millions of hospitalized patients are seriously affected by incurable strains of *A. baumannii*^37^. As an infection control measure, continuous studies on the resistance profile of this organism are highly required and are a must for at least decreasing its devastating effect on the quality of medical treatment^4^. In the current study, the prevalence of resistance among the 100 recovered *A. baumannii* isolates against the tested antimicrobial agents was high. A resistance prevalence of ≥ 78% was recorded for the tested isolates against the 19 antimicrobial agents used**.** On the other hand, colistin proved to be the most effective anti-microbial agent against these isolates (95% of isolates were sensitive) followed by doxycycline (43% of isolates were sensitive). Our finding agrees with previous studies which stated that, *A. baumannii* pathogen is an opportunistic organism, often susceptible to colistin and having a low susceptibility to other antimicrobial agents. This organism is involved in radical morbidity and mortality^38^.

The emerged pan drug-resistant (PDR), extensively drug-resistant (XDR)-*A. baumannii* strains could be a leading cause of hospital-acquired infections by this opportunistic pathogen^4^. PDR and XDR are being recorded increasingly among *A. baumannii* isolates recovered from clinical ^4, 39^ or environmental (such as soil) specimens^40^. In our finding, two isolates (2%) were detected as PDR, 68% isolates were XDR and 30% isolates were MDR**.** PDR and most of XDR- *A. baumannii* were isolated from Assiut university hospitals. The prevalence of MDR, XDR, and PDR *A. baumannii* could be attributed to the misuse of antimicrobial agents^41, 42^, or due to the differences in rates of infections by the respective pathogens (mostly related to the degree of strict hygiene protocols applied in different hospitals)^43^, in addition to the plasticity and endless capacity of changes demonstrated in *A. baumannii* genomes^44^.

Genotyping method using ERIC-PCR technique was applied for determining the clonal relationship and diversity of isolated *A. baumannii* used in the present study. This technique can be used during nosocomial outbreaks to investigate if the involved isolates are genetically related or originated from the same strains^45^. The use of strain typing in infectious disease control decisions in hospitals is based on several assumptions, (i) whether the isolates associated with an outbreak are the progeny of a single clone, or (ii) have identical genotype, or (ii) epidemiologically unrelated so have different genotypes^46^. In our study, the phylogenetic dendrogram of ERIC-PCR showed that the isolates can be divided into three major clusters. This diversity might be due to multiple contamination sources by this organism, a finding that is different from that reported by some authors^47^ who showed clonal expansion and microbial colonization by the *Acinetobacter baumannii* isolates used in their study. The obtained results necessitate the continuous monitoring of emerged genotypes among bacterial species implicated in hospital acquired infection and the development of new infection control strategies to comate the spread of such pathogens.

In the current study, investigation of the mechanisms responsible for the resistance of *A. baumannii* isolates to FQs involved detection of target-site mutation occurrence and acquisition of PAFQR genes. The analyses of target site mutations in *gyrA* and *parC* gene sequences of twelve selected *A. baumannii* isolates showed 12 profiles. Two isolates were susceptible to CIP and had a wild-type profile of being have no mutations. Ten isolates were CIP-resistant, 9 of them (9/10; 90%) had (1 *gyrA* /1 *parC*) mutation: Ser 81 → Leu mutation for *gyrA* gene and Ser 84 → Leu mutation for *parC* gene. The remaining CIP-resistant isolate (1/10; 10%) had (0 *gyrA* /1 *parC*) mutation: Ser 84 → Leu mutation for *parC* gene as reported previously in North Egypt^48^. Previous studies stated that double mutations (1*gyrA*/1*parC*) could be sufficient to confer CIP resistance in *A. baumannii* isolates^23, 48, 49^ and this in agreement with our results. . It was reported that these two mutations are enough to predict resistance to CIP and LEV^48^. However, *gyrA* and *parC* mutations did not occur in all resistant mutant strains and the resistance may be attributed to changes in outer membrane protein expression and drug efflux pumps^50^. All tested isolates had a silent mutation in one or more positions of either *gyrA* or *parC* or even both *gyrA* and *parC* that do not lead to a change in amino acid composition. Twenty-three isogenic mutations in *gyrA* genes were as follows: (6 Gly112, 7 Ala115, 4 Ile166, 4 Ala170, 2 Asp197) and eleven isogenic mutations in *parC* gene as follows: (2 Ala 52, 6 Leu 35, 1 Ala 127, 1 Gly 143, 1 Ala163). The isogenic mutations were identified previously in Gram-negative^51–53^, Gram-positive^54, 55^, *Mycobacterium*^56^, *Mycoplasma*^57^, and *A. baumannii*^58^. Our findings showed that four amino acid affected by isogenic mutations were detected in *gyrA,* these included Ala, Asp, Gly, and Ile while the three amino acid-affected by isogenic mutations in *parC* included Ala, Gly, and Ile and occurred in different codon positions. A storm of silent mutations was identified previously in *A. baumannii*^50^. Isogenic or silent mutations may be due to that the patient's administrated insufficient doses of FQs in different periods which may induce bacterial resistance to the drug^58^.

The dissemination of mobile elements harboring resistance genes between *Acinetobacter* spp. is not fully understood. Many earlier investigations have given special emphasis to plasmid-mediated horizontal transfer of antibiotic resistance genes^59–61^. In the present study, 99%; 99/100 of *A. baumannii* have harbored plasmids. However, one isolate that represents 1% has not harbored any plasmids. Despite plasmids themselves may be insufficient to confer FQs resistance, PAFQR genes execute an important role in the procuration of resistance to FQs by facilitating the selection of additional chromosomal resistance mechanisms, leading to a higher level of quinolone resistance and enabling bacteria to become fully resistant^62^. Most importantly, PAFQR genes can spread horizontally among *A. baumannii*^10^. Resistance to quinolones can be mediated by plasmids^63^. Three kinds of PAFQR determinants have been described: *qnr* genes (*qnrA, qnrB*, and *qnrS*) that encode pentapeptide protein repeats, which protect the quinolone targets from inhibition^8, 63^. Inactivation of fluoroquinolones occurs by acetylation with the common aminoglycoside acetyltransferase *aac (6*′*)-Ib-cr*^11^ and can be pumped out by efflux pumps QepAB and OqxAB^12^. The first PAFQR gene type is *qnr* genes, the 86, ciprofloxacin-resistant isolates carried *qnrA* (66.27%; 57/86), *qnrS* (70.93%; 61/86), while *qnrB* was undetected in these isolates. Such a high prevalence reported here and in other studies performed worldwide among FQ-resistant *A. baumannii* isolates reflects their fetal role in the acquisition of FQs resistance genes. In disparity, the lower prevalence of *qnr* was reported in CIP-resistance *A. baumannii* isolates from Brazil (37.5%) and north Egypt 48.3%^64^. On the other hand, *qnr* genes were more frequently detected among the isolates of *K*. *pneumoniae* (70.4%) and *E. coli* (67.5%)^61, 62, 65, 66^. The second PAFQR gene type is the *aac(6')-Ib-cr* gene, a new variant of common aminoglycoside acetyltransferase that acetylates piperazinyl substituent of some fluoroquinolones, including ciprofloxacin^11^. The bifunctional aminoglycoside and fluoroquinolone active variant *aac (6')-Ib-cr* catalyzes the acetylation of both drug classes^67^. PCR screening of isolates tested in the present study showed the prevalence of *aac (6')-Ib-cr* (52.32%; 45/86) among CIP-resistance isolates. On the other hand, CIP-susceptible isolates harbored *aac (6')-Ib-cr* by only 21.42% (3/14), these isolates were already resistant to aminoglycosides. Our result agrees those obtained in Iran and Brazil^68, 69^ but disagrees with that of Hamed et al.^25^ in north Egypt. The third PAFQR gene types are those encoding the efflux pumps QepA, and oqxAB^12^. QepA is a proton-dependent transporter belonging to the major facilitator superfamily that causes hydrophilic quinolone resistance^12^, while OqxAB is a transmissible resistance-nodulation-division multidrug efflux pump that was found to reduce susceptibility to CIP and nalidixic acid^70^. PCR screening showed the prevalence of *oqxA* by 73.25% (63/86) and *oqxB* by 39.53% (34/86) while *qepA* was undetected in these isolates. Our results don’t agree with that reported by Hamed et al.^25^ since they were unable to detect *oqxAB* in *A. baumannii* and *E. coli* while they could detect both in *Klebsiella* spp^25^. *qepA* was not detected in *A. baumannii , E. coli,* and *K. pneumoniae* but detected in *Enterobacter* spp. in the study conducted by^71^. Summing up, high variation in the prevalence of PAFQR efflux genes among different microbial species significantly limits the treatment options of infected patients and provides a potential source for the horizontal spread of resistance^71^. The differences mentioned above can result from the geographical distance, surveillance strategies, and variability in following up antibiotic stewardship among organizations.

Our findings showed that, although some of *A. baumannii* isolates (14%; 14/100) were CIP-sensitive, they harbored PAFQR genes: *qnrA* (7/14), *qnrS* (7/14), *aac (6')-Ib*-*cr* (3/14), *oqxA* (12/14), and *oqxB* (11/14) with collective number reached five PAFQR genes per a single isolate. This finding agrees with the results reported by other authors who showed that imipenem sensitive *A. baumannii* isolates harbored certain resistance genes^72, 73^ and the same was exhibited by other members of gram-negative bacilli worldwide^74^. The occurrence of resistance genes in CIP-sensitive *A. baumannii* isolates represents a major problem as this could facilitates their horizontal transfer between *A. baumannii* and other members of gram-negative bacilli in hospitals.

Much earlier theory has given special affirmation for transferable antibiotic resistance among *A. baumannii* by plasmid-associated quinolone resistance determinant genes^13, 59, 60^. Depending on the sequence data analysis of PAFQR gene amplicons conducted in our study, it was demonstrated that *oqxA* and *oqxB* were identified previously in *Klebsiella aerogenes* strain NCTC9793 and *Klebsiella pneumoniae*, respectively with identity of 99.77%. While, the sequences of *qnrS,* and *acc(6,)-ib-cr,* were identified previously in *A. baumannii* with identity ranged of 98.28% and 97.98%, respectively. Our results agree that of Hamed et al. in the north Egypt area who reported that *oqxAB* was not detected in *A. baumannii* and *E. coli* while it was detected in *Klebsiella* spp^25^. This result suggests the possibility of acquisition of transferable antibiotic resistance genes that may be carried on plasmids by *A. baumannii*.

Our results showed the existence of several PAFQR genes and their co-occurrence in *A. baumannii* recovered isolates. The distribution of tested PAFQR genes gave 26 profiles for the 86 CIP-resistant- and 8 profiles for the 14 CIP-sensitive isolates (both harbor up to five genes). The storm of association due to suppleness and the non-limit capacity of *A. baumannii* for genome changes^75^, which may be caused by horizontal gene transfer^76^, the introduction of mobile genetic elements like plasmids which mediate new genes, integrative conjugative elements, and transposons^60, 76^. These properties give rise to PDR, XDR, and MDR gene cassettes^77^.

## Conclusion

MDR, XDR, and PDR *A. baumannii* isolates are becoming prevalent in a number of hospitals, among the reasons behind this spread could be due to the empirical and un-rational use of antimicrobial agents which usually occur among outpatients in addition to improper infection control applied measures. It was observed that chromosomal mutations in the sequences of GyrA and ParC encoding genes as well as the acquisition of PAFQR encoding genes are molecular resistance mechanisms demonstrated among fluoroquinolones resistant *A. baumannii* isolates. It is advisable to monitor the antimicrobial resistance profiles of pathogens causing nosocomial infections and properly apply and update antibiotic stewardship in hospitals and for outpatients to control infectious diseases and prevent development of the microbial resistance to antimicrobial agents.

## Supplementary Information

Supplementary Information.
