# Supplementary material for: Impact of target site mutations and plasmid associated resistance genes acquisition on resistance of Acinetobacter baumannii to fluoroquinolones
Source: Sci Rep. 2021 Oct 11;11:20136. doi: 10.1038/s41598-021-99230-y (PMC8505613; doi:10.1038/s41598-021-99230-y)
Supplement: Supplementary file 1 — Supplementary Information. [file 41598_2021_99230_MOESM1_ESM.docx]

**
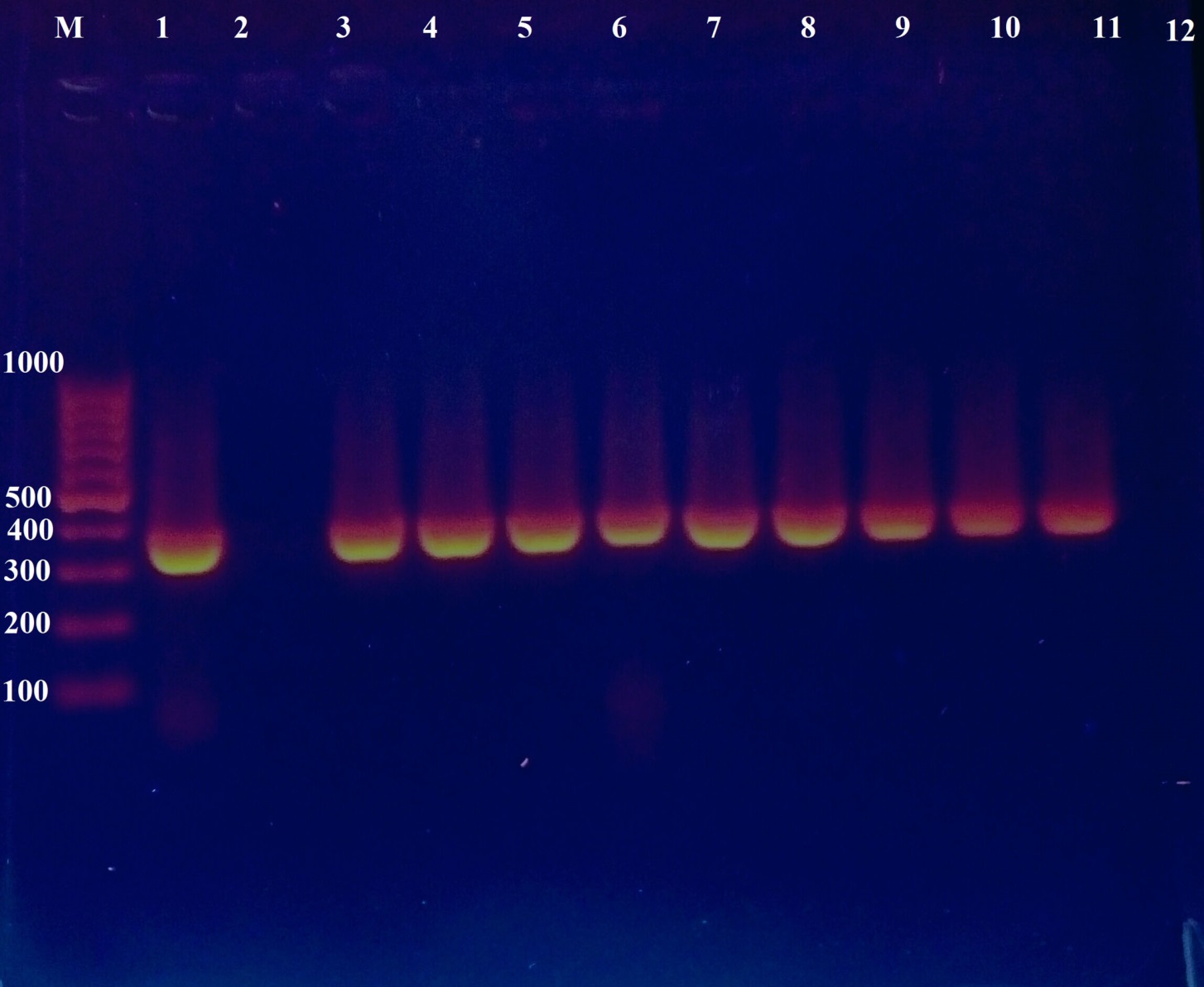
**

**Figure S1.** Agarose gel electrophoresis of PCR amplification products of *bla*_OXA-51_ like gene for some representative *A. baumannii* isolates. Lane M, a gene Ruler 100 bp ladder; lane 1, a positive control; lane 2, a negative control; lanes 3 to 11, positive results with an expected size of 353 bp; lane 12, negative results.

**Table** **S1.** Accession numbers of NCBI deposited sequences of *gyrA* and *parC* amplified fragments from selected *A. baumannii* isolates. AS: Isolates recovered from Assiut University hospitals, AZ: Isolates recovered from Al-Azhar University hospital

| Profile No. | Isolate code | ***GyrA*** | | ***ParC*** | |
| --- | --- | --- | --- | --- | --- |
|  |  | Gene Bank Accession No. | % of identity | Gene Bank Accession No. | % of identity |
| 1 | AS-31 | [CP054302.1](https://www.ncbi.nlm.nih.gov/nucleotide/CP054302.1?report=genbank&log$=nucltop&blast_rank=1&RID=MXE1FKHA014) | 99.83 | [CP041148.1](https://www.ncbi.nlm.nih.gov/nucleotide/CP041148.1?report=genbank&log$=nucltop&blast_rank=1&RID=MXFWR8ZT014) | 99.16 |
| 2 | AS-32 | [CP031380.1](https://www.ncbi.nlm.nih.gov/nucleotide/CP031380.1?report=genbank&log$=nucltop&blast_rank=1&RID=MXEDGBAD014) | 100 | [CP050385.1](https://www.ncbi.nlm.nih.gov/nucleotide/CP050385.1?report=genbank&log$=nucltop&blast_rank=1&RID=MXH1TVG8014) | 94.77 |
| 3 | AS-49 | [CP054302.1](https://www.ncbi.nlm.nih.gov/nucleotide/CP054302.1?report=genbank&log$=nucltop&blast_rank=1&RID=MXENGZ32014) | 100 | [CP041148.1](https://www.ncbi.nlm.nih.gov/nucleotide/CP041148.1?report=genbank&log$=nucltop&blast_rank=1&RID=MXH6PC3F014) | 99.03 |
| 4 | AZ-39 | [CP010397.1](https://www.ncbi.nlm.nih.gov/nucleotide/CP010397.1?report=genbank&log$=nucltop&blast_rank=1&RID=MXEHV6XS014) | 99.83 | [CP041148.1](https://www.ncbi.nlm.nih.gov/nucleotide/CP041148.1?report=genbank&log$=nucltop&blast_rank=1&RID=MXG1SDHD014) | 99.21 |
| 5 | AZ-13 | [CP051866.1](https://www.ncbi.nlm.nih.gov/nucleotide/CP051866.1?report=genbank&log$=nucltop&blast_rank=1&RID=MXET783P014) | 99.82 | [CP041148.1](https://www.ncbi.nlm.nih.gov/nucleotide/CP041148.1?report=genbank&log$=nucltop&blast_rank=1&RID=MXFR557Z014) | 99.03 |
| 6 | AS-28 | [CP054302.1](https://www.ncbi.nlm.nih.gov/nucleotide/CP054302.1?report=genbank&log$=nucltop&blast_rank=1&RID=MXF172KF01R) | 99.30 | [CP050385.1](https://www.ncbi.nlm.nih.gov/nucleotide/CP050385.1?report=genbank&log$=nucltop&blast_rank=1&RID=MXGXB7FH014) | 96.57 |
| 7 | AS-01 | [CP051862.1](https://www.ncbi.nlm.nih.gov/nucleotide/CP051862.1?report=genbank&log$=nucltop&blast_rank=1&RID=MXDX8S5X014) | 99.13 | [CP027246.2](https://www.ncbi.nlm.nih.gov/nucleotide/CP027246.2?report=genbank&log$=nucltop&blast_rank=1&RID=MXGTP34C016) | 98.25 |
| 8 | AS-29 | [CP054302.1](https://www.ncbi.nlm.nih.gov/nucleotide/CP054302.1?report=genbank&log$=nucltop&blast_rank=1&RID=MXF5EJJE014) | 99.83 | [CP041148.1](https://www.ncbi.nlm.nih.gov/nucleotide/CP041148.1?report=genbank&log$=nucltop&blast_rank=1&RID=MXGD9P0S014) | 98.87 |
| 9 | AZ-01 | [CP054302.1](https://www.ncbi.nlm.nih.gov/nucleotide/CP054302.1?report=genbank&log$=nucltop&blast_rank=1&RID=MXFGFTUE014) | 96.91 | [CP027246.2](https://www.ncbi.nlm.nih.gov/nucleotide/CP027246.2?report=genbank&log$=nucltop&blast_rank=1&RID=MXGPC0XD014) | 98.07 |
| 10 | AZ-33 | [CP051866.1](https://www.ncbi.nlm.nih.gov/nucleotide/CP051866.1?report=genbank&log$=nucltop&blast_rank=1&RID=MXFA3ZH2014) | 100 | [CP041148.1](https://www.ncbi.nlm.nih.gov/nucleotide/CP041148.1?report=genbank&log$=nucltop&blast_rank=1&RID=MXGJFFJ9016) | 99.03 |
| 11 | AS-13 | [CP040050.1](https://www.ncbi.nlm.nih.gov/nucleotide/CP040050.1?report=genbank&log$=nucltop&blast_rank=1&RID=MXFD2W0N014) | 99.61 | [CP027246.2](https://www.ncbi.nlm.nih.gov/nucleotide/CP027246.2?report=genbank&log$=nucltop&blast_rank=1&RID=MXG8GU1M014) | 98.16 |
| 12 | AS-05 | [CP032055.1](https://www.ncbi.nlm.nih.gov/nucleotide/CP032055.1?report=genbank&log$=nucltop&blast_rank=1&RID=MXEVXP4E014) | 99.47 | [CP027246.2](https://www.ncbi.nlm.nih.gov/nucleotide/CP027246.2?report=genbank&log$=nucltop&blast_rank=1&RID=MXG4WUHJ014) | 95.59 |

**Table** S**2.** Quinolone resistance mutations in the *gyrA* and *ParC* genes of *A. baumannii* clinical isolates

| **Isolate code** | **Profile No. (No. of antimicrobial agents)*** | ***GyrA* mutation** | | |  | ***ParC*mutation** | | |
| --- | --- | --- | --- | --- | --- | --- | --- | --- |
|  |  | **I^st^** | **2^nd^** | **3^rd^** | **4^th^** | **I^st^** | **2^nd^** | **3^rd^** |
| AS-31 | 1 (19) | Ser 81(TCA) | Gly 112 (GGC) | Ala 115 (GCC) | WT | Ala 52 (GCC) | Ser 84 (TCG) | WT |
|  |  | Leu 81 (TTA) | Gly 112 (GGT) | Ala 115 (GCT) |  | Ala 52 (GCT) | Leu 84 (TTG) |  |
|  |  | Substitution | Silent | Silent |  | Silent | Substitution |  |
| AS-32 | 2 (18) | Ser 81(TCA) | Gly 112 (GGC) | Ala 115 (GCC) | WT | Leu 35 (TTG) | Ser 84 (TCG) | WT |
|  |  | Leu 81 (TTA) | Gly 112 (GGT) | Ala 115 (GCT) |  | Leu 35 (TTA) | Leu 84 (TTG) |  |
|  |  | Substitution | Silent | Silent |  | Silent | Substitution |  |
| AS-49 | 3 (17) | Ser 81(TCA) | Gly 112 (GGC) | Ala 115 (GCC) | WT | Leu 35 (TTG) | Ser 84 (TCG) | WT |
|  |  | Leu 81 (TTA) | Gly 112 (GGT) | Ala 115 (GCT) |  | Leu 35 (TTA) | Leu 84 (TTG) |  |
|  |  | Substitution | Silent | Silent |  | Silent | Substitution |  |
| AZ-39 | 4 (16) | Gly 112 (GGC) | Ala 115 (GCC) | - | WT | Serine 84 (TCG) | WT | |
|  |  | Gly 112 (GGT) | Ala 115 (GCT) |  |  | Leu 84 (TTG) |  |  |
|  |  | Silent | Silent |  |  | Substitution |  |  |
| AZ-13 | 5 (15) | Ser 81(TCA) | Ile166 (ATC) | Ala170 (GCG) | WT | Leu 35 (TTG) | Ser 84 (TCG) | WT |
|  |  | Leu 81 (TTA) | Ile 166 (ATT) | Ala 170(GCT) |  | Leu 35 (TTA) | Leu 84 (TTG) |  |
|  |  | Substitution | Silent | Silent |  | Silent | Substitution |  |
| AZ-28 | 6 (14) | Ser 81(TCA) | Ile166 (ATC) | Ala170 (GCG) | Asp 197 (GAT) | Ser 84 (TCG) | WT | |
|  |  | Leu 81 (TTA) | Ile 166 (ATT) | Ala 170(GCT) | Asp 197 (GAC) | Leu 84 (TTG) |  |  |
|  |  | Substitution | Silent | Silent | Silent | Substitution |  |  |
| AS-01© | 7 (13) | WT | | | | Ala 127 (GCC) | Gly 143 (GGT) | Ala 163 (GCA) |
|  |  |  |  |  |  | Ala 127 (GCA) | Gly 143 (GGC) | Ala 163 (GCG) |
|  |  |  |  |  |  | Silent | Silent | Silent |
| AS-29 | 8 (12) | Ser 81(TCA) | Gly 112 (GGC) | Ala 115 (GCC) | WT | Ala 52 (GCC) | Ser 84 (TCG) | WT |
|  |  | Leu 81 (TTA) | Gly 112 (GGT) | Ala 115 (GCT) |  | Ala 52 (GCT) | Leu 84 (TTG) |  |
|  |  | Substitution | Silent | Silent |  | Silent | Substitution |  |
| AZ-01 | 9 (11) | Ser 81(TCA) | WT | | | Leu 35 (TTG) | Ser 84 (TCG) | WT |
|  |  | Leu 81 (TTA) |  |  |  | Leu 35 (TTA) | Leu 84 (TTG) |  |
|  |  | Substitution |  |  |  | Silent | Substitution |  |
| AZ-33 | 10 (09) | Ser 81(TCA) | Ile166 (ATC) | Ala170 (GCG) | Asp 197 (GAT) | Leu 35 (TTG) | Ser 84 (TCG) | WT |
|  |  | Leu 81 (TTA) | Ile 166 (ATT) | Ala 170(GCT) | Asp 197 (GAC) | Leu 35 (TTA) | Leu 84 (TTG) |  |
|  |  | Substitution | Silent | Silent | Silent | Silent | Substitution |  |
| AS-13 | 11 (08) | Ser 81(TCA) | Gly 112 (GGC) | Ala 115 (GCC) | WT | Leu 35 (TTG) | Ser 84 (TCG) | WT |
|  |  | Leu 81 (TTA) | Gly 112 (GGT) | Ala 115 (GCT) |  | Leu 35 (TTA) | Leu 84 (TTG) |  |
|  |  | Substitution | Silent | Silent |  | Silent | Substitution |  |
| AS-05^(c)^ | 12 (06) | Ala 115 (GCC) | Ile166 (ATC) | Ala170 (GCG) | WT | WT | | |
|  |  | Ala 115 (GCT) | Ile 166 (ATT) | Ala 170(GCT) |  |  |  |  |
|  |  | Silent | Silent | Silent |  |  |  |  |

WT, Wild-type; MICs, Minimum inhibitory concentrations; AS, isolates recovered from Assiut University hospitals; AZ, isolates recovered from Al-Azhar University hospitals.

***** To which the tested isolates were resistant

^(c)^ To which the tested isolates were CIP-sensitive while the others were CIP-resistant
